# Mutations in DNA repair genes are associated with increased neo-antigen load and activated T cell infiltration in lung adenocarcinoma

**DOI:** 10.18632/oncotarget.23742

**Published:** 2017-12-15

**Authors:** Young Kwang Chae, Jonathan F. Anker, Preeti Bais, Sandeep Namburi, Francis J. Giles, Jeffrey H. Chuang

**Affiliations:** ^1^ Northwestern University Feinberg School of Medicine, Chicago, 60611, IL, USA; ^2^ Robert H. Lurie Comprehensive Cancer Center of Northwestern University, Chicago, 60611, IL, USA; ^3^ The Jackson Laboratory for Genomic Medicine, Farmington, 06030, CT, USA; ^4^ Department of Genetics and Genome Sciences, University of Connecticut Health, Farmington, 06032, CT, USA

**Keywords:** DNA repair, tumor mutational burden, neo-antigens, lung cancer, tumor infiltrating lymphocytes

## Abstract

Mutations in DNA repair genes lead to increased genomic instability and mutation frequency. These mutations represent potential biomarkers for cancer immunotherapy efficacy, as high tumor mutational burden has been associated with increased neo-antigens and tumor infiltrating lymphocytes. While mismatch repair mutations have successfully predicted response to anti-PD-1 therapy in colorectal and other cancers, they have not yet been tested for lung cancer, and few have investigated genes from other DNA repair pathways. We utilized TCGA samples to comprehensively immunophenotype lung tumors and analyze the links between DNA repair mutations, neo-antigen and total mutational burden, and tumor immune infiltration. Overall, 73% of lung tumors contained infiltration by at least one T cell subset, with high mutational burden tumors containing significantly increased infiltration by activated CD4 and CD8 T cells. Further, mutations in mismatch repair genes, homologous recombination genes, or POLE accurately predicted increased tumor mutational burden, neo-antigen load, and T cell infiltration. Finally, neo-antigen load correlated with expression of M1-polarized macrophage genes, PD-1, PD-L1, IFNγ, GZMB, and FASLG, among other immune-related genes. Overall, after defining the immune infiltrate in lung tumors, we demonstrate the potential value of utilizing gene mutations from multiple DNA repair pathways as biomarkers for lung cancer immunotherapy.

## INTRODUCTION

Recent studies have uncovered that tumor types with high somatic mutation frequencies, such as non-small cell lung cancer (NSCLCs), melanoma, and colorectal cancer (CRC) [1], display the strongest responses to immune checkpoint inhibitors [2–7]. This link between mutational burden and immunotherapy efficacy is due to the generation of immunogenic, tumor-specific mutated peptides, termed neo-antigens. Neo-antigens have been linked to response to anti-PD-1 therapy in NSCLC [8] and anti-CTLA-4 therapy in melanoma [9]. Further, in muscle invasive bladder cancer, high neo-antigen load correlated with greater recurrence-free survival and low T cell receptor-β diversity, indicating oligoclonal T cell expansion [10]. Across multiple cancer types, predicted neo-antigen burden, but not overall mutational load, correlated with patient survival and *CD8A* and *PD-1* expression [11], as well as immune cytolytic activity (*GZMA* and *PRF1* expression) [12].

The clinical significance of neo-antigens is derived from their ability to drive a functional and specific anti-tumor immune response. Neo-antigen-specific T cells have been identified in chronic lymphocytic leukemia patients who demonstrated remission after allogeneic stem cell therapy [13], melanoma patients who were administered an IL-12-producing dendritic cell vaccine (after prior ipilimumab therapy) [14], as well as phase II melanoma patients who experienced tumor regression after adoptive transfer of autologous tumor infiltrating lymphocytes (TILs) [15]. In pre-clinical studies, multiple immunogenic mutated peptides capable of decreasing tumor growth *in vivo* were identified in the B16F10 melanoma cells line [16], and in a murine sarcoma model, tumor immunoediting led to clonal outgrowth of tumor cells lacking neo-antigen expression [17]. Further, immunization with neo-antigens has even proven therapeutically comparable to administration of immune checkpoint blockade in mice [18]. This therapeutic potential was translated to the clinical setting, as adoptive transfer of autologous tumor mutation-specific Th1 CD4 T cells from a patient with metastatic cholangiocarcinoma was able to induce tumor regression upon original administration as well as after tumor recurrence [19].

One potential driver of a hypermutable state capable of generating neo-antigens is the loss of DNA repair genes [20]. In CRC, mismatch repair (MMR) gene mutations differentiate microsatellite instable (MSI) from microsatellite stable (MSS) disease. Interestingly, MSI CRCs contain increased mutations, predicted neo-antigens, TILs (with a Th1 phenotype), and expression of immunosuppressive molecules, and MSI also confers a favorable prognosis [21–27]. Mutated MMR genes themselves have even been postulated as immunogenic antigens capable of driving the immune response in pancreatic cancer [28]. Importantly, in a phase 2 trial of metastatic CRC patients treated with the anti-PD-1 antibody, pembrolizumab, patients with MMR-deficient tumors had a objective response rate (ORR) of 40%, compared to 0% for those with MMR-proficient tumors. Similarly, patients with MMR-deficient non-CRC had a ORR of 71%. These MMR-deficient tumors contained significantly increased mutation and neo-antigen load, both of which were associated with increased progression-free survival [6]. Importantly, similar success has been replicated in MSI-high CRC [29] and across multiple other cancer types [30], leading to pembrolizumab being granted FDA approval for use in unresectable or metastatic MMR-deficient or MSI-high solid tumors. However, none of the above studies have demonstrated efficacy in MMR-deficient lung tumors, and immunotherapy clinical trials have only focused on the MMR DNA repair pathway.

In addition to mutations in MMR genes, deficiencies in other DNA repair genes have also been implicated in neo-antigen generation and impacting the immune response. *BRCA1/2* and other homologous recombination (HR) gene mutations have been associated with increased neo-antigen load, CD3 and CD8 TILs, expression of cytotoxicity-related genes (in the TCR, IFNγ, and TNFR pathways), *PD-1* and *PD-L1* expression, and favorable overall survival (OS) in ovarian cancer [31, 32]. In endometrial cancer, samples with *POLE* mutations have shown increased neo-antigens, as well as *PD-1* and *PD-L1* expression [33]. Further, in NSCLC patients treated with anti-PD-1 blockade, responders with the highest mutational burden contained mutations in *POLD1, POLE,* and *MSH2,* and nonsynonymous mutational burden and neo-antigen load in these patients correlated with enhanced clinical response [8]. This effect of DNA repair loss to augment the anti-tumor immune response can explain the improved clinical outcomes and survival seen in patients with tumors harboring mutations in these critical genes [34–36].

In lung adenocarcinoma, nivolumab immunotherapy is approved for patients progressing on platinum-based chemotherapy, conferring a 51% 1 year OS rate compared to 39% with docetaxel [37]. Additionally, pembrolizumab is approved for PD-L1-positive metastatic NSCLC patients, with a reported 19.4% ORR and 12 month median OS (treated population was 81% non-squamous NSCLC) [5]. However, while neo-antigen and mutational burden have been linked to enhanced response to immunotherapies [8], biomarkers to aid in identifying these patients are lacking. In addition, the overall immunophenotype of the lung adenocarcinoma microenvironment still has not clearly been defined in a comprehensive manner. We hypothesize that mutations in DNA repair genes in lung adenocarcinoma are key players in determining total mutational burden, neo-antigen load, and, consequently, tumor microenvironment in increased TILs and immune infiltrate that can serve as future biomarkers for immune checkpoint efficacy.

## RESULTS

### The immunophenotype of lung adenocarcinoma is primarily infiltration by activated CD4 and CD8 T cells

**Figure 1:**
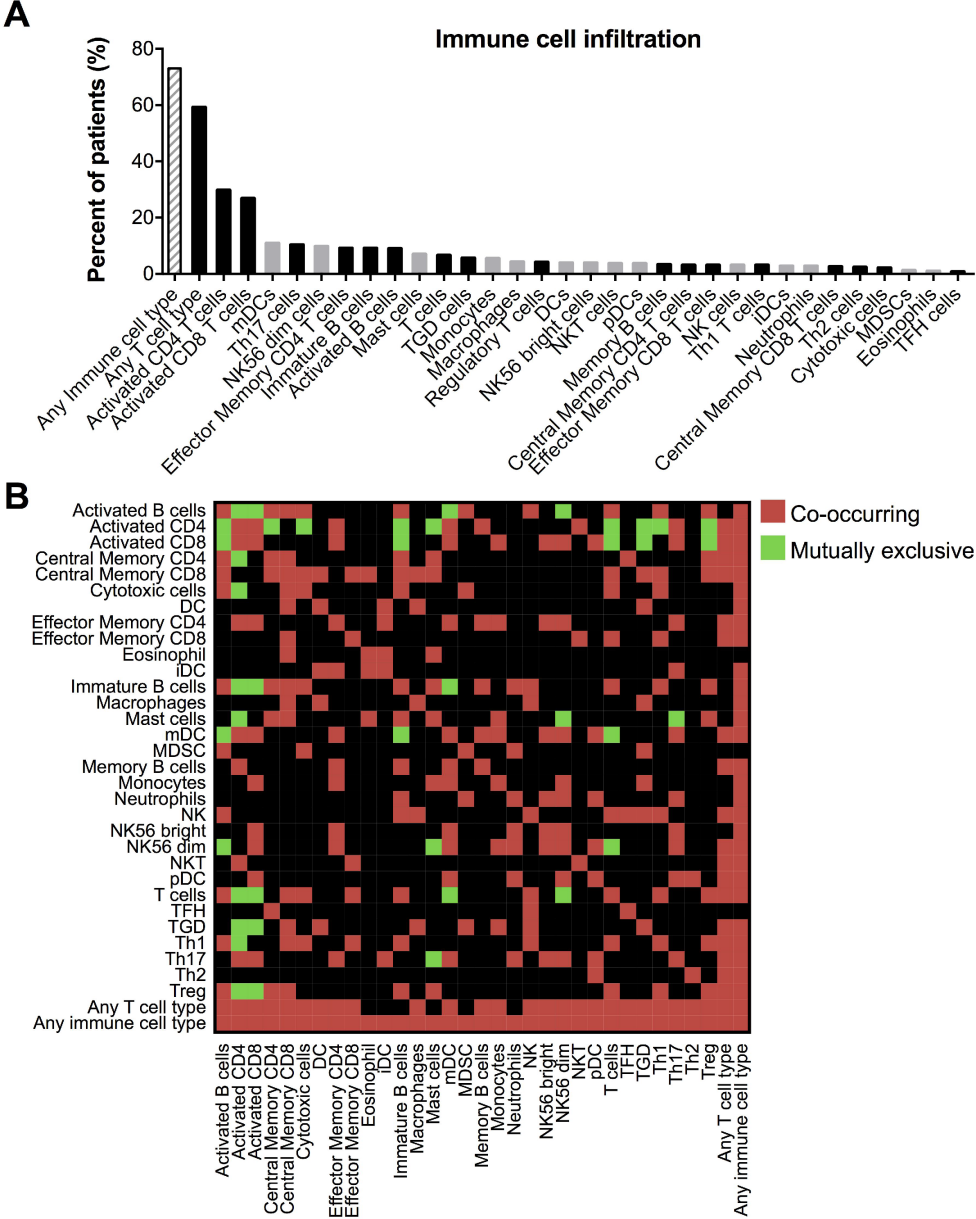
Comprehensive immunophenotype of the lung adenocarcinoma microenvironment (**A)** Percent of tumors with positive infiltration by each adaptive (black bars) and innate (gray bars) immune cell type, as determined by immune metagene lists and GSEA analysis. Abbreviations: γδ T (TGD) cells, immature DCs (iDCs), myeloid derived suppressor cells (MDSCs), follicular helper T (TFH) cells. (**B)** Heat map indicating infiltration of immune cell types that either significantly (^*^P < 0.05) co-occur or are mutually exclusive within tumors.

To elucidate the immune microenvironment of TCGA lung adenocarcinoma samples, we utilized a pre-determined list of immune metagenes whose expression have been shown to accurately predict the infiltration of 28 immune cell populations [24]. Activated CD4 and activated CD8 T cells were the most prominent infiltrating cell types, present in 29.7% and 26.8% of samples, respectively. Infiltration by at least one T cell subtype was identified in 73% of samples (Figure 1A). Additionally, we analyzed the tendency for each pairing of immune cell types to infiltrate the same samples. Interestingly, infiltration by activated CD4 and CD8 T cells significantly co-occurred in the same tumors, and infiltration by both cell types also significantly co-occurred with myeloid dendritic cells (mDCs), effector memory CD4 T cells, and Th17 T cells, with both inversely correlating with activated B cells, immature B cells, regulatory T cells (Tregs) and γδ T cells (Figure 1B).

### Tumors with higher mutational burden contain increased TILs and a distinct molecular and histological phenotype

**Figure 2:**
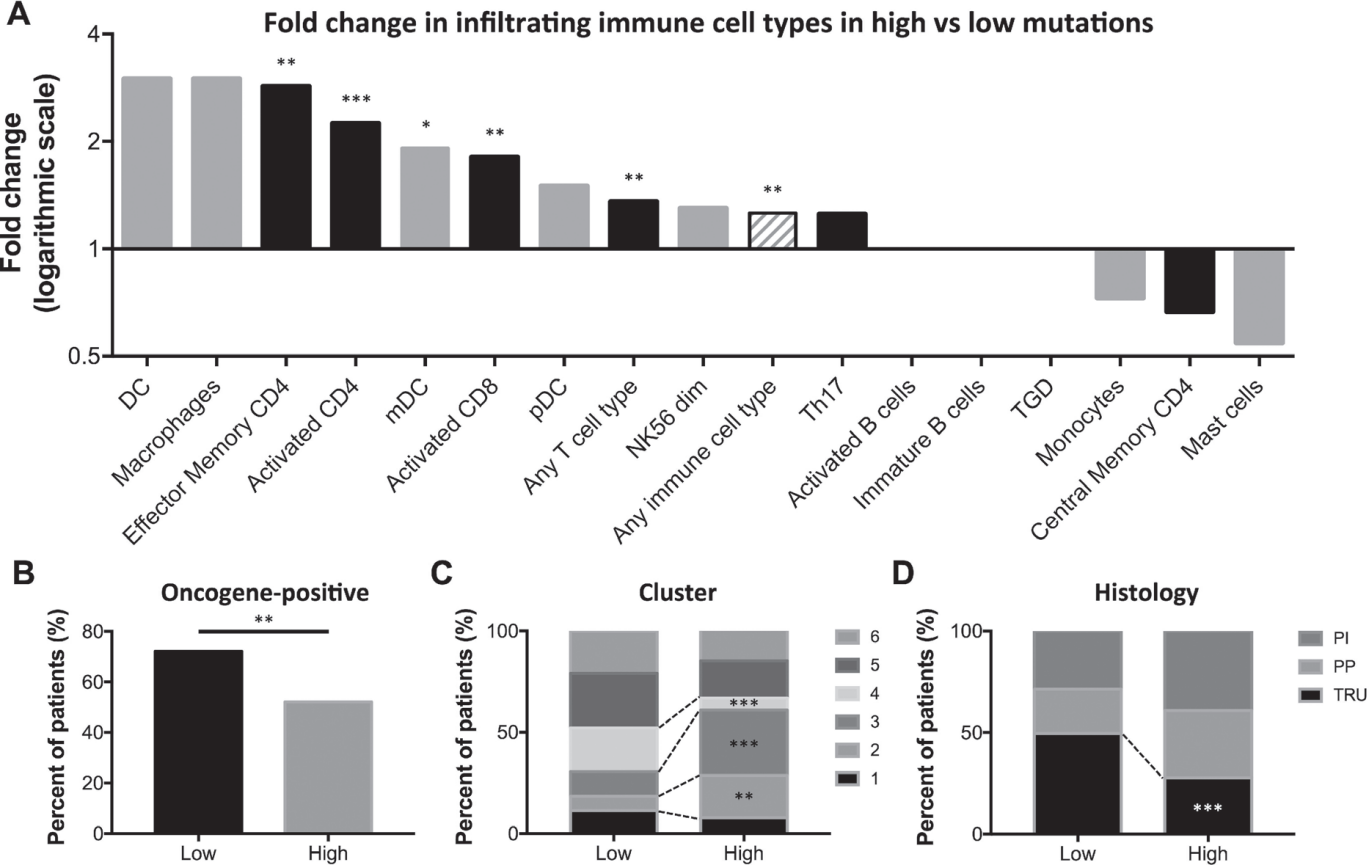
High mutation burden tumors contain increased TILS, immune infiltration, and a distinct molecular phenotype (**A**) Fold change in the percent of patients with adaptive (black bars) and innate (gray bars) immune cell infiltration in those with high compared to low mutation burden, as defined by the median mutation count (excluding cell types with positive tumor infiltration in < 10 samples). Percent of high and low mutational burden tumors classified by (**B**) oncogene-positivity, (**C**) cluster type, and (**D**) histology. ^*^ *P* < 0.05, ^**^ *P* < 0.01, ^***^ *P* < 0.001.

Mutational burden is known to impact tumor immunogenicity. To determine how the immunophenotype of lung adenocarcinoma is affected by increased mutational burden, we divided samples into those above or below the median mutation count. As expected, patients with tumors with high mutational burden were heavier smokers (Supplementary Figure 1A, 1B) and contained increased cytosine to adenine transversions (Supplementary Figure 1C), which has been previously linked to smoking [38–40]. Interestingly, the high mutation group also contained significantly higher infiltration by effector memory CD4 T cells, activated CD4 and CD8 T cells, mDCs, any T cell type, and any immune cell type (Figure 2A). Additionally, tumors with higher mutational burden were less likely to be oncogene-positive, as defined by containing an activating mutation in the RTK/RAS/RAF signaling pathway (Figure 2B). These tumors were also associated with a cluster 2 or 3 classification, defined by increased *TP53* mutations, tumor ploidy, and copy number variations (CNVs) in 8q, 15q, and chromosome 7. High mutation tumors were less commonly cluster 4 samples, which are defined by low ploidy, 6q CNVs, *CDKN2A* methylation, and *SETD2* mutations (Figure 2C). Finally, high mutation tumors were more often histologically proximal-inflammatory (squamoid; *NF1* and *TP53* mutations) or proximal proliferative (magnoid; *KRAS* mutation, *STK11* inactivation), and significantly less likely to be terminal respiratory unit (bronchioid; *EFGR* mutations and kinase fusions) [38] (Figure 2D).

### DNA repair-deficient tumors contain increased mutation count, TILs

**Figure 3:**
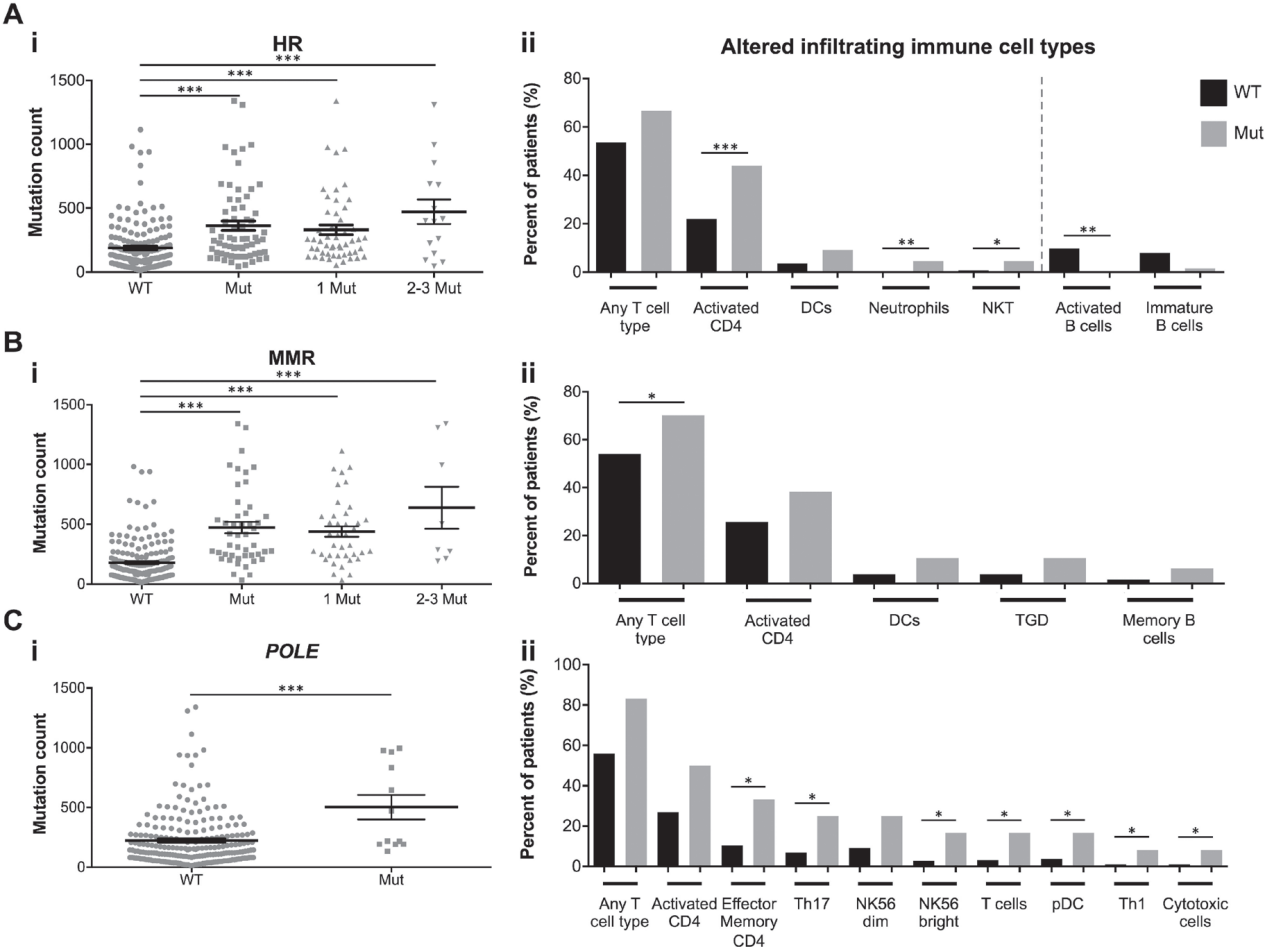
DNA repair mutations are biomarkers for total mutational burden and TILs Total mutation count of tumors with mutations in (**A.**i) HR genes, (**B.**i) MMR genes, or (**C.**i) *POLE.* Results are presented as mean ± SEM. (**A.**ii), (**B**.ii), (**C**.ii) Percent of DNA repair wildtype (WT; black bars) or mutated (Mut; gray bars) tumors with immune cell infiltration (all immune cell types with *P* < 0.1 displayed. Red dotted line separates increased and decreased tumor infiltrating immune cell types). ^*^ *P* < 0.05, ^**^ *P* < 0.01, ^***^ *P* < 0.001.

Mutations in DNA repair genes can consequently result in a hypermutable state with increased genomic instability. To test our hypothesis that mutations in these single DNA repair genes can serve as biomarkers for identifying NSCLC patients with higher mutational burden and increased TILs, we analyzed tumors specifically containing mutations in the HR pathway, the MMR pathway, or *POLE*. In each grouping, tumors with DNA repair deficiencies displayed significantly increased mutation counts (and a trending further increased mutation count with multiple HR and MMR genes mutated) (Figure 3A.i, 3B.i, 3C.i). Samples with HR mutations were significantly more infiltrated by activated CD4 T cells, neutrophils, natural killer T (NKT) cells, and were less likely to be infiltrated by activated B cells. Those with MMR mutations contained increased T cell infiltration, and *POLE* mutated samples contained increased infiltration by effector memory CD4 T cells, Th17 T cells, natural killer (NK)56 bright cells, T cells, plasmacytoid DCs (pDCs), Th1 T cells, and cytotoxic cells (Figure 3A.ii, 3B.ii, 3C.ii).

We next analyzed the link between DNA repair gene mutations and mutations in type I interferon (IFN) genes, as tumor-derived type I IFNs are important for optimal immunosurveillance and anti-cancer therapy efficacy [41]. Samples with HR, MMR, and *POLE* mutations all contained increased frequencies of mutations in various type I IFN pathway genes. Notable, HR mutated tumors contained increased IFNβ (*IFNB1*) and *IRF8* (type I IFN positive feedback loop) mutations, while MMR mutated tumors contained increased IFNα (*IFNA5*, *IFNA14*, *IFNA21)* and *IFNAR2* (type I IFN receptor) mutations (Supplementary Figure 2A–2C), thereby indicating that while mutations in DNA repair genes are linked to increased tumor mutation count and TILs, they also may represent tumors with increased type I IFN gene mutations. Similarly, we also analyzed potential immune evasion as a result of mutations in class I HLA genes [42]. However, class I HLA mutations in the TCGA dataset we rare, with only 3, 0, and 1 of the 230 samples containing mutations in *HLA-A*, *HLA-B*, and *HLA-C*, respectively. Of those, the 3 *HLA-A* mutated samples did not contain HR, MMR, or *POLE* mutations, while the single *HLA-C* mutated sample did contain a mutated HR pathway (data not shown).

### Neo-antigen burden is linked to DNA repair mutations, increased TILs

**Figure 4:**
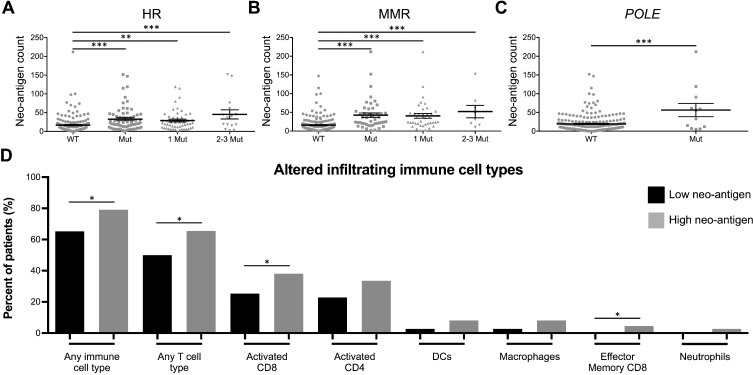
DNA repair mutations are biomarkers for high tumor neo-antigen load, which is associated with increased TILs Neo-antigen count of tumors with (**A)** HR, (**B**) MMR, or (**C)** *POLE* mutations. Results are presented as mean ± SEM. (**D**) Percent of neo-antigen low (black bars) or high (gray bars) patients infiltrated by immune cell types (all immune cell types with *P* < 0.1 displayed). ^*^ *P* < 0.05, ^**^ *P* < 0.01, ^***^ *P* < 0.001.

The clinical and immunological relevance of increased mutational burden is that as more tumor mutations arise, there is a greater likelihood for somatic, non-synonymous mutations to result in the formation of immunogenic epitopes expressed only in cancerous cells. Therefore, we filtered total non-synonymous mutations into predicted neo-antigens, as determined by predicted immunogenicity (MHC binding affinity) (Supplementary Figure 3A), positive gene expression (Supplementary Figure 3B), and those whose non-mutated parental epitope were weak or non-MHC binders (Supplementary Figure 3C). Similar to total mutation count, neo-antigen burden was also significantly higher in samples with HR, MMR, or *POLE* mutations (Figure 4A–4C). Additionally, those samples with high neo-antigen load contained significantly increased infiltration by any immune cell type, overall T cells, activated CD8 T cells, and effector memory CD8 T cells (Figure 4D).

### Neo-antigens correlate with increased expression of multiple pro-inflammatory cytokines and immune-related genes, M1-polarized macrophage genes, *PD-L1* and *PD-1*, and favorable survival outcome

**Figure 5:**
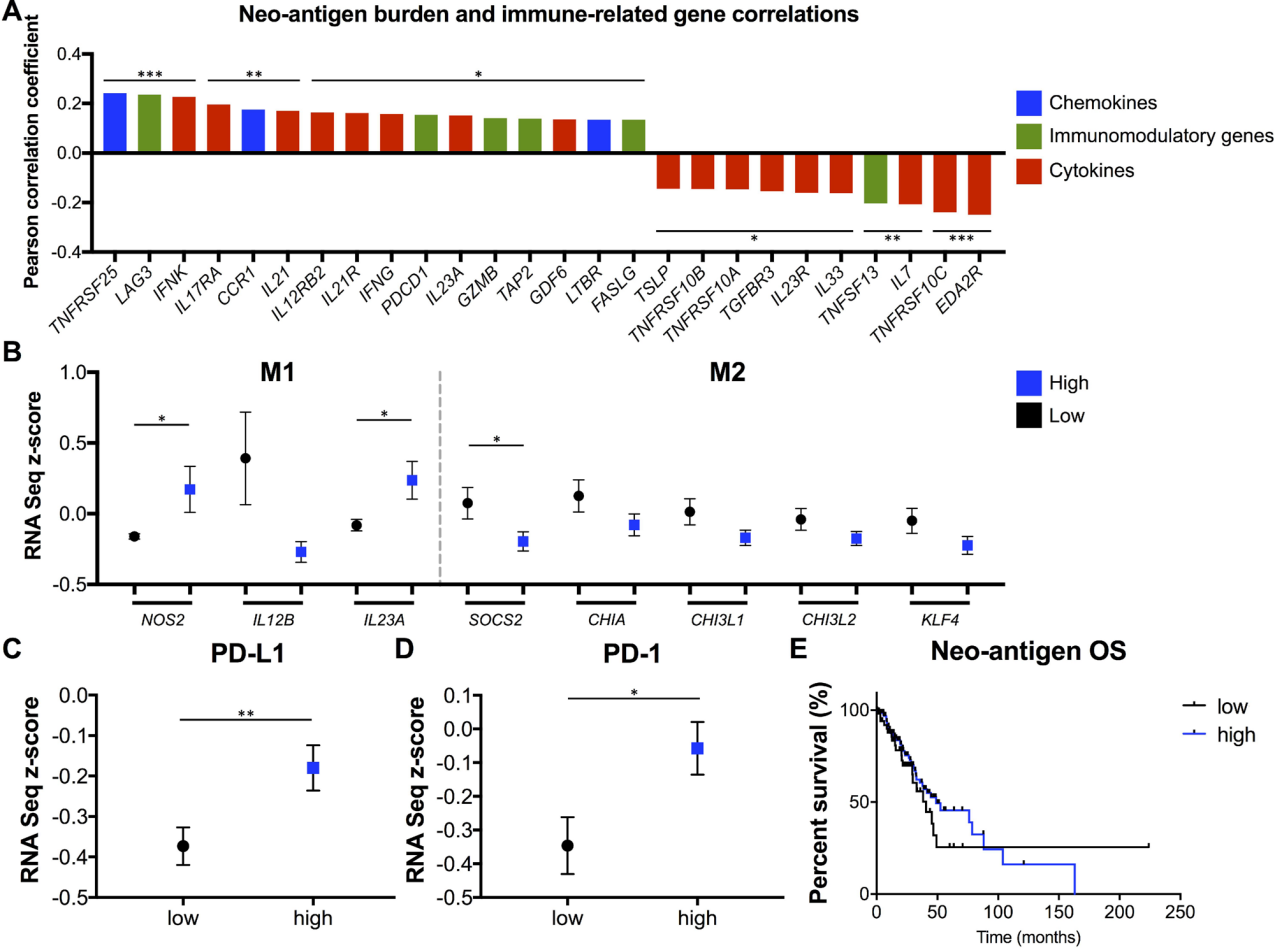
Neo-antigen load correlates with multiple immune-related genes and cytokines, M1-polarized TAMs, *PD-L1*, *PD-1*, and favorable OS (**A)** Pearson correlation coefficients between neo-antigen count and RNA-seq expression levels of multiple chemokines, immunomodulatory genes, and cytokines (only significantly correlating genes reported). (**B**) Expression of M1 TAM- and M2 TAM-defining genes in high and low neo-antigen burden tumors (all genes with *P* < 0.2 displayed). (**C**) *PD-L1* and (**D**) *PD-1* expression in high (top quartile) and low neo-antigen load tumors. Results are presented as mean ± SEM. (**E**) OS of neo-antigen load high (top quartile) and low tumors. ^*^ *P* < 0.05, ^**^ *P* < 0.01, ^***^ *P* < 0.001.

While the link between neo-antigen load and TILs is now appreciated in many cancer types, the specific molecular causes and consequences of that association are not as well understood. We analyzed the correlations between neo-antigen burden and the expression of a comprehensive list of cytokines, chemokines, and immunomodulatory genes [24]. Significant positively or negatively correlated genes are reported in Figure 5A. Increasing neo-antigen load correlated with chemokines *TNFRSF25, CCR1,* and *LTBR*, immunomodulatory genes *LAG3, PDCD1 (PD-1), GZMB,* and *FASLG*, and multiple cytokines/cytokine receptors, including *IFN*γ, *IL12RB2,* and *IL17RA.* Additionally, when analyzing expression of gene markers to differentiate pro-inflammatory M1 from immunosuppressive M2 tumor-associated macrophages (TAMs) [43], tumors with more neo-antigens displayed significantly increased expression of M1 genes *NOS2* and *IL23A,* and significantly (*SOC2*) or trending (*CHIA, CHI3L1, CHI3L2, KLF4*) decreased expression of multiple M2-associated genes (Figure 5B). Further, the top quartile of high neo-antigen load samples displayed increased *PD-L1* (Figure 5C) and *PD-1* (Figure 5D) expression. Finally, these high neo-antigen load patients trended toward increased OS (*p* = 0.12 when excluding the low neo-antigen outlier censored at 224 months) (Figure 5E).

## DISCUSSION

Recent scientific and clinical advances in cancer immunotherapy have highlighted the importance of understanding neo-antigens and the immunological tumor microenvironment. Specifically in lung cancer, immune checkpoint blockade has shown great promise. However, we are still lacking a comprehensive understanding of the immunophenotype of these tumors. Additionally, while deficiencies in DNA repair genes, mostly in the MMR pathway, are known to accelerate mutation rates and affect prognosis, their relevance in immunotherapy in lung cancer has been unclear.

We first analyzed immune cell infiltration, utilizing a comprehensive list of “immune metagenes” to accurately predict the presence of up to 28 intra-tumoral immune cell types in a large cohort of lung adenocarcinoma patients. Interestingly, activated CD4 and CD8 T cells were the most commonly infiltrating cell types, followed by mDCs, all of which are desired for an optimal anti-tumor response. 73% of the samples were infiltrated by at least one of the T cell subtypes (Figure 1A). Further, activated CD4 and CD8 T cells were low in tumors infiltrated by activated B cells or immature B cells (Figure 1B), in agreement with a study demonstrating increased CD8 tumor infiltration and decreased tumor growth after B cell depletion [44]. Activated CD8 and CD4 infiltrated tumors also contained decreased infiltration by Tregs but increased Th17 T cell infiltration. Differentiation of T cells into either of these cell types inhibits the generation of the other, yet while Tregs are known to promote immunosuppression and tumor growth [45], intra-tumoral Th17 T cells can increase IFNγ production and tumor infiltration by CD8 TILs, NK cells, and DCs [46]. Also of note, tumors infiltrated by activated CD4 and CD8 T cells also contained increased infiltration by γδ T cells, which can also promote the anti-tumor immune response [47].

We next sought to determine the significance of mutational burden on the immunological response in lung adenocarcinoma. As hypothesized, tumors with high mutational count displayed increased infiltration by activated CD4 and CD8 T cells, effector memory CD4 T cells, and mDCs (Figure 2A). Importantly, tumors containing mutations in HR genes, MMR genes, or *POLE* contained significantly higher mutational load than DNA repair wildtype tumors, and similarly contained significantly increased infiltration by many of the T cell types and other immune cells desirable for anti-tumor activity. Further, HR-deficient tumors also contained decreased infiltration by B cells, a potentially immunosuppressive and tumor-promoting cell type [48] (Figure 3). Therefore, detecting mutations in the single *POLE* gene or the small HR or MMR gene lists can accurately be used as biomarkers for identifying patients whose tumors likely contain high mutational burden and, consequently, increased TILs.

To clarify the clinical relevance of these findings, we refined overall mutational burden to specifically consider at predicted immunogenic neo-antigens. HR-, MMR-, and *POLE*-deficient tumors again contained increased neo-antigens and T cell infiltration (Figure 4). Interestingly, increasing neo-antigen load strongly positively correlated with expression of many immune-related genes. Notably, neo-antigens increased with increasing expression of *IFN*γ and *IL12RB2*, indicating a Th1 skewed signature, as is desirable in an anti-tumor response. Neo-antigen load also correlated with increased expression of *IL23A*, *IL21*, and *IL17RA*, all of which are players in the Th17 T cell pathway, which is implicated in both tumorigenesis as well as the anti-tumor immune response [46]. Also correlating were *GZMB* and *FASLG*, both necessary for anti-tumor CD8 T cell cytotoxic functionality [49], *PD-1* and *LAG3*, representing T cell activation and exhaustion [50], and *TAP2*, necessary in MHC I antigen presentation [51]. Cytokines inversely correlating with neo-antigen burden included *TSLP* and *IL33*, involved in the Th2 response [52], *IL7*, implicated with pro- and anti-tumorigenic properties [53], *EDA2R*, which is involved in p53 signaling [53], and multiple genes in the TRAIL apoptosis pathway. Additionally, neo-antigen load correlated with increased expression of the chemokines *TNFRSF25*, *CCR1*, and *LTBR*, which may serve as valuable targets in future tumor immunology studies (Figure 5A).

Outside of T cells, TAMs have emerged an important focus of research and therapeutic targets. Specifically, M1-polarized macrophages have been shown to promote anti-tumor activity and decrease tumor growth while M2 macrophages suppress TILs and promote tumorigenesis [54]. We found that high neo-antigen load correlated with increased expression of multiple M1 genes and decreased expression of multiple M2 genes. This finding supports a novel link between neo-antigens and M1 TAM polarization. This may be due to the interplay between an increased activated adaptive T cell response with a Th1/Th17 signature and the infiltrating innate cells. Finally, tumors with increased neo-antigens contained increased expression of *PD-L1* (Figure 5C), which is induced by IFNγ [55], and PD-1 (Figure 5D), as well as a trending increase in OS (Figure 5E). While past studies have reported a more dramatic gap in survival between high and low neo-antigen groups, our analysis was limited because for the TCGA data the treatments and disease stage were not controlled between groups, many patients were censored at early time points, and many patients had resectable early stage disease as opposed to late stage disease important for survival analysis.

Overall, we comprehensively analyzed the immunophenotype of the lung adenocarcinoma microenvironment. This study demonstrates that lung tumors with DNA repair deficiencies, either in the MMR pathway, HR pathway, or *POLE,* can be utilized as biomarkers for total mutational burden, neo-antigen load, and TILs, which will be of utmost value as immunotherapies continue to grow and expand clinically. These results provide a foundation for further studies exploring the clinical implications of using DNA repair gene mutations as a predictive marker for immunotherapy response.

## MATERIALS AND METHODS

### Clinical data, immune infiltration, DNA repair

This study utilized cBioPortal [56, 57] to obtain data from the lung adenocarcinoma cohort of The Cancer Genome Atlas (TCGA) [38] to analyze RNA-sequencing (RNA-seq) gene expression and clinical data (*n* = 515) and DNA mutations (*n* = 230). Predicted infiltration by 28 distinct immune cell types was performed as previously described [24], utilizing 812 “immune metagenes” derived from 813 microarrays over 36 studies. Expression of these genes was used as input in Gene Set Enrichment Analysis (GSEA), and any immune cell types with a false discovery rate (*q*-value) of ≤ 10% were considered as positively infiltrating into that tumor sample. In the DNA repair genes analysis, the homologous recombination pathway gene list included *ATR, ATM, CHEK1, CHEK2, BRCA1, BRCA2, BAP1, BARD1, FANCD2, FANCE, FANCC, FANCA, RAD50, RAD51,* and *PALB2*, and the mismatch repair pathway gene list included *MLH1, MLH3, MSH2, MSH3, MSH4, MSH5, MSH6, PMS1, PMS2, PMS2L3, PCNA, EXO1, POLD1, RFC1, RFC2, RFC3, RFC4,* and *RFC5* [58]. In the type I IFN signaling pathway analysis, the gene list included all genes with available TCGA data from the gene ontology (GO) accession number 0060337 [59, 60].

### Neo-antigen prediction

HLA typing, neo-antigen identification, and HLA-peptide affinity prediction were performed using HLAminer [61], Variant Effect Predictor Tool [62], NetMHCpan [63], and the UCSC browser [64] (http://www.genome.ucsc.edu/), with most computations performed on the National Cancer Institute (NCI) Cancer Genomics Cloud (CGC) in the Seven Bridges Genomics implementation. The neo-antigen affinity scores were generated between all possible 9 amino acid length peptides containing a mutated site with the 6 predicted HLA types, using the CloudNeo pipeline [65]. A control analysis was performed with the homologous non-mutated 9 amino acid length sequences. Neo-antigens were defined as mutated peptides with a binding score IC_50_ < 500 nM, positive gene expression, and whose non-mutated wildtype peptide was a weak or non-MHC binder IC_50_ > 500 nM.

### Statistical analysis

As appropriate, z-score between two population proportions, unpaired two-tailed Student's or Welch's *t-*test, Log-rank test for survival, and the Pearson correlation coefficient were used for statistical assessment. Results are presented as percentages, fold change (logarithmic scale), mean ± standard error of the mean (SEM), Pearson correlation coefficient, RNA-seq z-score, or percent survival, as indicated. ^*^ *P* < 0.05, ^**^ *P* < 0.01, ^***^ *P* < 0.001.

## SUPPLEMENTARY MATERIALS FIGURES



## Abbreviations

NSCLC: non-small cell lung cancer
CRC: colorectal cancer
TIL: tumor infiltrating lymphocyte
MMR: mismatch repair
MSI: microsatellite instable
MSS: microsatellite stable
ORR: objective response rate
HR: homologous recombination
OS: overall survival
mDC: myeloid dendritic cell
Treg: regulatory T cells
CNV: copy number variations
TAM: tumor-associated macrophage
TCGA: The Cancer Genome Atlas
GSEA: Gene Set Enrichment Analysis
CGC: Cancer Genomics Cloud
SEM: standard error of the mean
TGD: γδ T cell
NKT: natural killer T cell
pDC: plasmacytoid DC
NK: natural killer cell
iDC: immature DC
MDSC: myeloid derived suppressor cell
TFH: follicular helper T cell
WT: wildtype
Mut: mutated
RNA-seq: RNA-sequencing
