# Supplementary material for: Mutations in DNA repair genes are associated with increased neo-antigen load and activated T cell infiltration in lung adenocarcinoma
Source: Oncotarget. 2017 Dec 15;9(8):7949–60. doi: 10.18632/oncotarget.23742 (PMC5814272; doi:10.18632/oncotarget.23742)
Supplement: Supplementary file 1 [file oncotarget-09-7949-s001.pdf]

Mutations in DNA repair genes are associated with increased neo-antigen load and activated T cell infiltration in lung adenocarcinoma

SUPPLEMENTARY MATERIALS

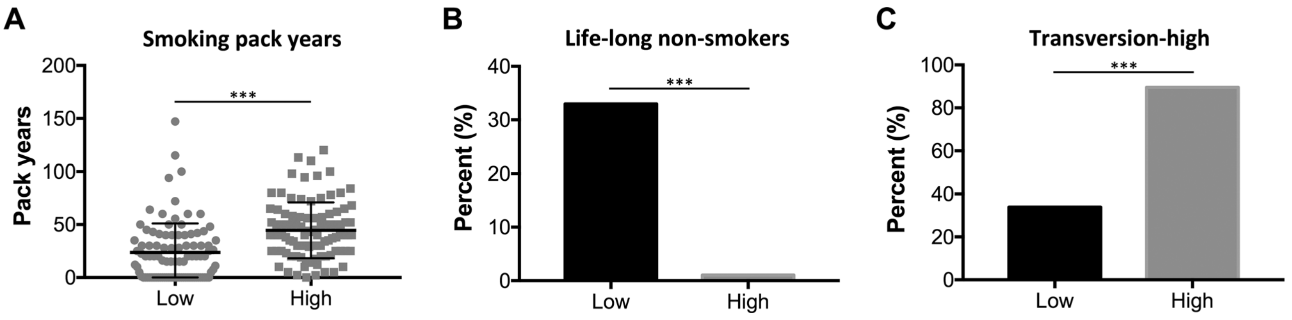

Supplementary Figure 1: Tumor mutational burden is increased in smokers and contains increased transversions. High and low total mutational burden tumors classified by (**A**) patient smoking pack years (results are presented as mean ± SEM), (**B**) percent of life-long non-smokers, and (**C**) percent of transversion-high tumors. *** $P < 0.001$.

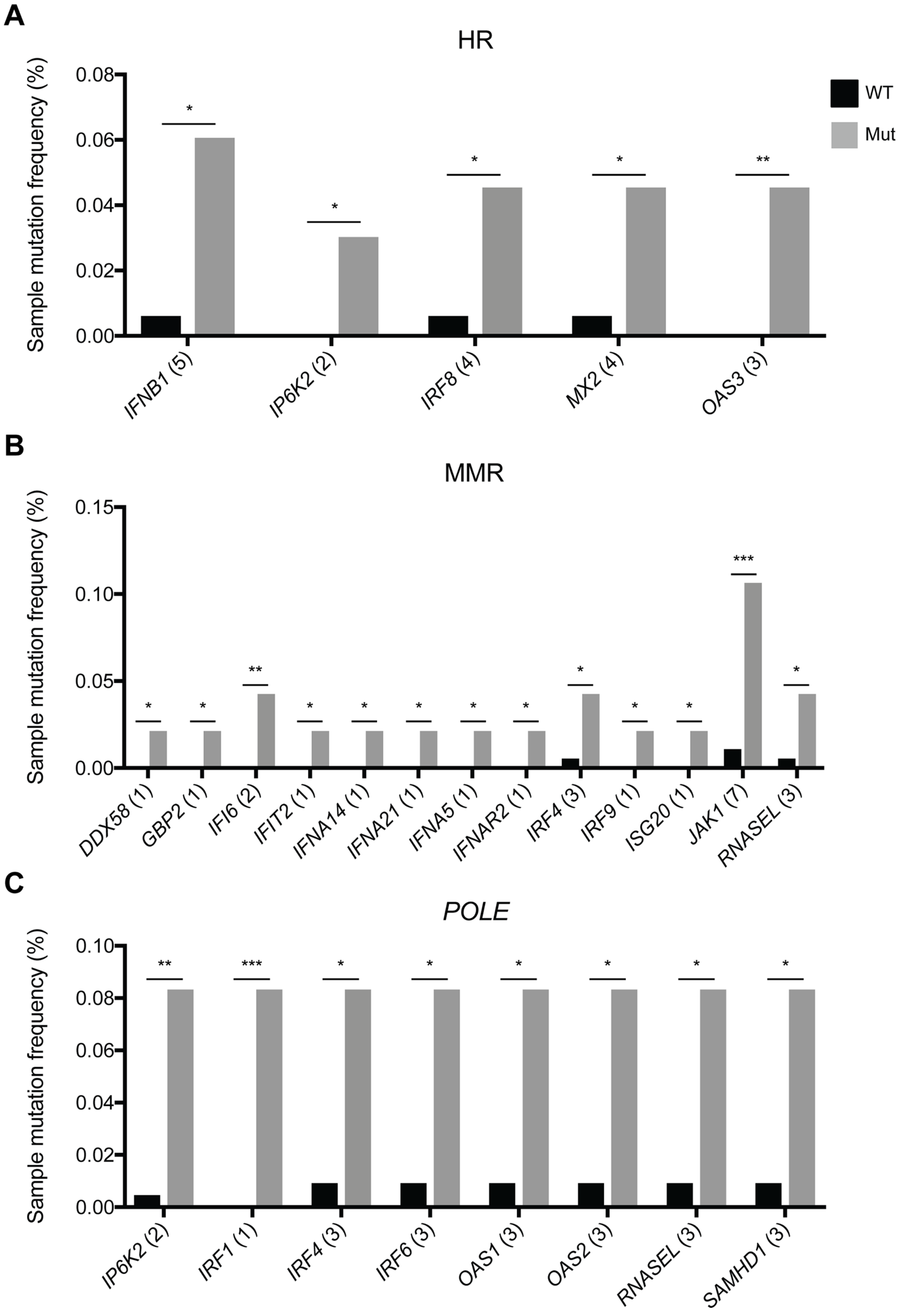

Supplementary Figure 2: DNA repair mutations are linked to increased frequencies of type I IFN gene mutations. The mutation frequency of all type I IFN signaling pathway genes (GO: 0060337) with mutation data from the TCGA dataset were analyzed in samples with WT or mutated (**A**) HR genes, (**B**) MMR genes, or (**C**) *POLE*. All genes with significantly altered mutation frequencies are displayed. Numbers in parenthesis indicate the number of samples which contained a mutation in each gene. * $P < 0.05$, ** $P < 0.01$, *** $P < 0.001$.

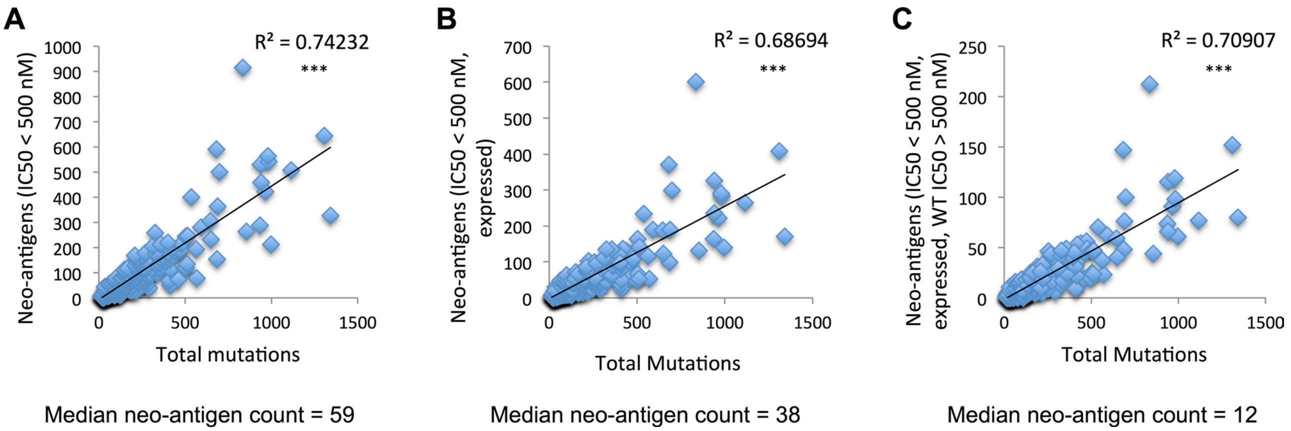

Supplementary Figure 3: Filters used in predicting neo-antigen load. Correlation between neo-antigen load and total mutation count as defined by (**A**) predicted MHC binding affinity (IC50 < 500 nM), (**B**) predicted MHC binding affinity (IC50 < 500 nM) and positive gene expression, and (**C**) predicted MHC binding affinity (IC50 < 500 nM), positive gene expression, and non-mutated parental peptide predicted MHC binding affinity (IC50 > 500 nM). *** $P < 0.001$.
